# Fluid Intelligence Predicts Novel Rule Implementation in a Distributed Frontoparietal Control Network

**DOI:** 10.1523/JNEUROSCI.2478-16.2017

**Published:** 2017-05-03

**Authors:** Nadja Tschentscher, Daniel Mitchell, John Duncan

**Affiliations:** ^1^Medical Research Council, Cognition and Brain Sciences Unit, Cambridge CB2 7EF, United Kingdom and; ^2^Max Planck Institute for Human Cognitive and Brain Sciences, 04103 Leipzig, Germany

**Keywords:** executive functions, fluid intelligence, fMRI, frontoparietal control system, goal-directed behavior

## Abstract

Fluid intelligence has been associated with a distributed cognitive control or multiple-demand (MD) network, comprising regions of lateral frontal, insular, dorsomedial frontal, and parietal cortex. Human fluid intelligence is also intimately linked to task complexity, and the process of solving complex problems in a sequence of simpler, more focused parts. Here, a complex target detection task included multiple independent rules, applied one at a time in successive task epochs. Although only one rule was applied at a time, increasing task complexity (i.e., the number of rules) impaired performance in participants of lower fluid intelligence. Accompanying this loss of performance was reduced response to rule-critical events across the distributed MD network. The results link fluid intelligence and MD function to a process of attentional focus on the successive parts of complex behavior.

**SIGNIFICANCE STATEMENT** Fluid intelligence is intimately linked to the ability to structure complex problems in a sequence of simpler, more focused parts. We examine the basis for this link in the functions of a distributed frontoparietal or multiple-demand (MD) network. With increased task complexity, participants of lower fluid intelligence showed reduced responses to task-critical events. Reduced responses in the MD system were accompanied by impaired behavioral performance. Low fluid intelligence is linked to poor foregrounding of task-critical information across a distributed MD system.

## Introduction

The neural basis of fluid intelligence is a fascinating but poorly understood topic. Lesion and imaging evidence links fluid intelligence to a specific set of frontal and parietal regions, here called the multiple-demand network (Prabhakaran et al., 1997; Duncan et al., 2000; Gray et al., 2003; Woolgar et al., 2010; Cole et al., 2015). This network includes the intraparietal sulcus, the anterior (ant)—posterior (post) axis of the lateral frontal cortex (LFC), the anterior insula (AI), the frontal eye field (FEF), and the pre-supplementary motor area (pre-SMA). Functional magnetic resonance imaging (fMRI) research demonstrates the recruitment of the multiple-demand network in many kinds of tasks, including tests of working memory, response inhibition, attention switching, and many more (Duncan, 2013; Fedorenko et al., 2013).

Usually, fluid intelligence is measured with complex, multistep tasks (Raven et al., 1988), and in novel behavior, errors linked to low fluid intelligence increase with the number of operations or rules involved in a task (Duncan et al., 2008; Bhandari and Duncan, 2014). Intriguingly, even rules that are known not to apply to a current trial, or block of trials, can increase errors on the remainder (Duncan et al., 2008), suggesting a broad inability to foreground the correct part of a complex rule set. A general requirement in complex behavior is to select and assemble the specific components of each task stage or epoch (Duncan, 2013). Achieving focus on the correct cognitive operations may become increasingly demanding with increases in total task complexity or the total body of potentially competing operations. Previously, we have proposed that achieving such focus may be a core aspect of multiple-demand function, accounting for widespread recruitment across many kinds of tasks (Duncan, 2013; Bhandari and Duncan, 2014).

Using fMRI, previous research showed that trial-by-trial changes in executive demands during a working memory task led to specific event-related fMRI responses in frontal-parietal cortex as a function of fluid intelligence levels (Gray et al., 2003). Higher fluid intelligence was associated with greater event-related neural activity for more challenging trials, which correlates with higher performance accuracies. Here we extended this work to examine the joint influence of fluid intelligence and contextual task complexity. Over different runs of the experiment, sets of novel rules were instructed. To manipulate task complexity, subjects memorized either two or four novel rules in different experimental runs, although in each 29 s epoch of performance only one rule applied. Although only one rule applied at a time, we predicted that participants with low fluid intelligence could have difficulty configuring multiple-demand activity for this rule, especially in the more complex, four-rule context. Accordingly, we predicted increased performance errors, accompanied by decreased multiple-demand responses to critical task events.

## Materials and Methods

### 

#### 

##### Participants.

Participants were prescreened and divided into two groups, a high-IQ group and a low-IQ group, based on their scores in a test of fluid intelligence (Cattell and Cattell, 1960). All 38 participants had an IQ score of at least 85. Two subjects were excluded because they failed to correctly recall the task rules. The study was approved by the Cambridge Psychology Research Ethics Committee, and participants gave informed consent and were paid for taking part. Participants had no history of psychological or neurological health problems and reported normal or corrected-to-normal vision. The mean age of participants was 44 years, ranging between 29 and 65 years. The mean IQ score from the Culture Fair Test (Cattell and Cattell, 1960) was 107 (SD = 16). There was no significant correlation between IQ scores and age in our subject sample (*p* = 0.120). Based on a median-split analysis of all IQ raw scores (median IQ = 105), we defined a low-IQ group as having scores smaller or equal to the median, and a high-IQ group as having scores larger than the median. The low-IQ group included 20 participants (8 females) with a mean IQ score of 95 (SD = 8). The high-IQ group included 16 participants (10 female) with a mean IQ score of 122 (SD = 10).

##### Experimental paradigm.

**Figure 1.**
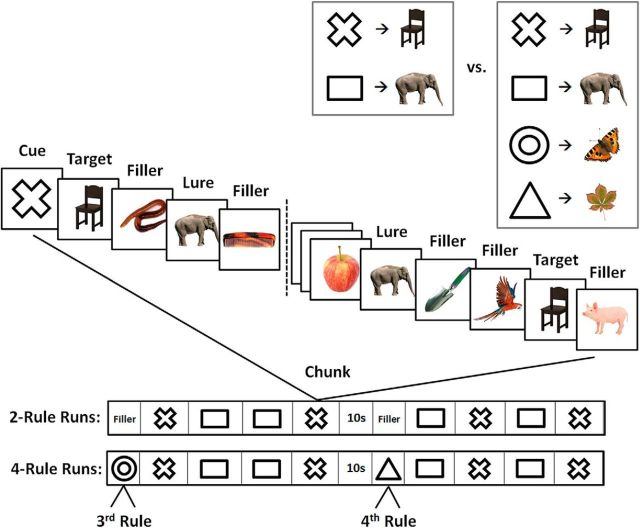
The experimental paradigm. Top, Before each experimental run, participants learned two or four novel cue–target associations. Middle, In each task chunk (29 s), a cue preceded a sequence of animate and inanimate objects. The cue indicated the target in the current chunk. Other images were fillers and lures (images associated with a different cue). Bottom, Sequence of task chunks in two-rule and four-rule runs.

The design of the task is illustrated in Figure 1. For each 4.5 min run of the experiment, participants were asked to memorize two or four novel associations between geometric figures (cues) and animate or inanimate objects (targets). The run was then divided into a series of chunks, each beginning with the appearance of a single cue from the memorized set, followed by a series of pictures presented at 1/s (0.7 s on, 0.3 s interstimulus interval). The task was to press a key with the right hand whenever the specified target appeared.

Each run (Fig. 1, bottom) was divided into two halves by a 10 s pause in the middle (mid). For two-rule runs, each half contained four 29 s chunks, two for each rule in ABAB, BABA, ABBA, or BAAB order. Within each chunk, the cued target appeared twice, as did the image associated with the other cue (lure, to be ignored). Remaining pictures were fillers, which were never used as targets, with the number of fillers between each critical event (cue, target, or lure) jittered between two and eight. There was a brief period (15 s) of filler pictures before the first chunk of each half-run, again presented at 1/s, which the participant simply watched while awaiting the first cue. For four-rule runs, two extra rules (secondary rules) were used only once each, one for the 15 s period at the start of the first half of the run, the other for the similar period at the start of the second half. These 15 s periods consisted of the cue, 12 fillers, and, randomly placed within these, a single target and a single lure (the image associated with the alternative secondary cue). The other two rules (primary rules) were used for remaining chunks, which were matched in organization and structure to the chunks of two-rule runs. To match two-rule runs, lures in these primary-rule chunks were always the image associated with the noncued primary rule. To ensure the comparability of two- and four-rule data, analysis focused just on the eight main chunks of each run, discarding the initial period of each half-run (fillers for two-rule runs, secondary rules for four-rule runs). Across the whole experiment there were 10 runs: 5 two-rule runs and 5 four-rule runs. Different cues and targets were used for each run. In two-rule runs, one target was animate and the other was inanimate. In four-rule runs, this was separately true for primary and secondary rules. One half of the filler items on each run was animate, the other half was inanimate.

Before the beginning of each run, two slides were presented. The first slide indicated the cue–target associations (Fig. 1, top, as an example), and stated “please memorize the following associations.” The second slide asked subjects to recall the images associated with the previously learned cues. The order of cues presented for the memory check was randomized and did not match the order of presentation on the initial instruction slide. Subjects stated their responses during the memory check verbally and were allowed to see the initial instruction slide again in case they were not confident. At the end of each run, another memory check slide was presented, asking subjects to recall the images associated with the randomly presented cues.

##### Stimuli and visual display.

Stimuli were presented using MATLAB Psychtoolbox-3 (Kleiner et al., 2007). Stimuli were colorful images of animals and objects drawn from multiple open-source visual image databases with a visual angle ranging from 3° to 4.7°. The visual display included a white background and a black fixation cross that was presented in the 0.3 s interstimulus interval.

##### Data acquisition.

fMRI data were acquired using a Siemens 3 T TimTrio Scanner with a 64-channel head coil. We used a standard echoplanar imaging sequence. Parameters were as follows: TR (interscan interval) = 2 s; TE = 30 ms; and flip angle = 78°. The functional images consisted of 32 slices covering the whole brain (slice thickness, 3 mm; interslice distance, 0.75 mm; in-plane resolution, 3 × 3 mm). A structural MPRAGE MRI (256 × 240 × 160, 1 mm isotropic) was acquired for all participants.

##### Data processing and statistical analysis.

The preprocessing and general linear modeling (GLM) were conducted using the automated analysis pipeline (version 4.0; Cusack et al., 2015) that was run in SPM12 (Wellcome Trust Centre for Neuroimaging, University College London, London, UK). First-level statistical contrasts were computed by using the GLM based on the canonical hemodynamic response function (Friston et al., 1998). Low-frequency noise was removed with a high-pass filter (time constant, 128 s). Events were separately modeled for each of the 10 runs. Within each run, the onsets and durations for correct cues, targets, lures, and filler items were separately modeled for each chunk. For this purpose, responses to targets were considered correct if they occurred in the 1 s following target onset (i.e., before onset of the following stimulus); responses to cues, lures, and fillers were considered correct if there was no keypress in this time period. Each run also contained one regressor modeling the duration of the two 15 s periods (fillers or secondary rules) at the start of each half-run, one regressor modeling the errors (i.e., false alarms for lures, fillers, and cues, as well as misses for targets), as well as six regressors for the movement parameters (the three parameters of translational and rotational movements, respectively). To eliminate chunks where participants may have missed a cue or searched for the wrong target, all targets and lures for a particular chunk were removed (modeled as errors) if no response was given to either of the two targets, an equal number of responses were given to targets and lures, or more responses were given to lures than to targets. Such error chunks occurred rarely, making up 1.7% of all chunks across runs and participants. These chunks were also excluded from behavioral analyses.

Contrasts for events were defined on a single-subject level first and then were subjected to random-effects analyses for group statistics using SPM12. Signal from predefined regions of interest (ROIs) of the multiple-demand network was extracted using the Marsbar utility in SPM12 (Brett et al., 2002). A template for multiple-demand network ROIs was used, defined in MNI space in a separate study that contrasted difficult and easy conditions across seven tasks in 40 participants (Fedorenko et al., 2013; see *t* map at http://imaging.mrc-cbu.cam.ac.uk/imaging/MDsystem). This network template (see Figs. 3, 4) included the intraparietal sulcus (IPS), the anterior–posterior axis of the LFC (ant-LFC, mid-LFC, and post-LFC), the AI, the FEF, and the pre-SMA. ROIs were symmetrically defined for the left and right hemispheres.

## Results

### Behavioral results

Data from 36 participants were analyzed. Two subjects were excluded because they either failed to memorize some of the task rules in the beginning of a run or failed to retrieve them at the end of a run. For all analyzed participants, accordingly, rule learning and retrieval were perfect.

**Figure 2.**
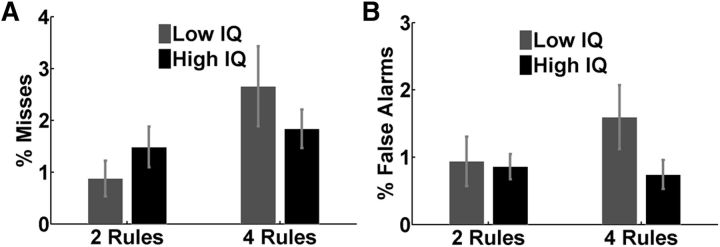
Behavioral results from the eight main chunks of all experimental runs. ***A***, Percentages of misses for targets. ***B***, Percentages of false alarms for lures. Error bars indicate SEM.

Our primary behavioral analyses concerned just the eight main chunks of each run, examining misses to targets (no keypress within 1 s of stimulus onset) and false alarms to lures (keypress in corresponding interval). The impact of task complexity and IQ was assessed by applying an ANOVA with the factors “complexity” (two vs four rules) and “IQ group” (high-IQ vs low-IQ). For misses, there was a significant main effect of complexity (*F*_(1,34)_ = 10.71, *p* = 0.002) as well as a significant complexity × IQ group interaction (*F*_(1,34)_ = 4.81, *p* = 0.035), which was driven by higher percentages of misses in the low-IQ group during four-rule runs (Fig. 2*A*). Trends were similar for false alarms, though in this case there were no significant main effects or interactions (Fig. 2*B*). No significant main effect of IQ group was observed. To evaluate the proportion of correct responses (hits) in relation to false alarms, we also calculated the sensitivity or *d*′ index (referring to *d*′ = *Z*(hit rate) − *Z*(false alarm rate; MacMillan and Creelman, 2005). This is a measure of overall performance accuracy (i.e., how well subjects distinguished between the two critical stimulus events; targets and lures). This analysis confirmed the main effect of complexity (*F*_(1,34)_ = 12.517, *p* = 0.001), as well as the complexity × IQ group interaction effect (*F*_(1,34)_ = 10.087, *p* = 0.003). The overall percentages of misses and false alarms were low, ranging between 1% and 4% across participants, indicating good overall ability to follow task instructions.

To ensure that all participants adequately considered all four rules in the four-rule conditions, we also examined misses and false alarms in the brief task periods devoted to the two secondary rules (Fig. 1). The number of missed targets was low, and did not differ significantly between IQ groups (mean percentage of misses for low-IQ = 5.5, SD = 1.7; mean percentage of misses for high-IQ = 5.0, SD = 1.5; *t*_(34)_ = 0.2, *p* = 0.85). The absence of significant IQ group effects might reflect the lack of statistical power inherent in analyses of the secondary rules, since those rules only appeared twice within each four-rule run. False alarm rates for secondary rules were also low, this time with a significant difference between groups (mean percentage of false alarms for low-IQ = 2.5, SD = 1; mean percentage of false alarms for high-IQ = 0; *t*_(34)_ = 2.24, *p* = 0.031). Overall, the results confirm that secondary rules, like primary rules, were learned and followed.

### fMRI results

We assessed the recruitment of the multiple-demand network during performance of the task by extracting univariate fMRI parameter estimates from seven predefined ROIs within each hemisphere (ant-LFC, mid-LFC, post-LFC, FEF, IPS, AI, and pre-SMA). Our main interest concerned the impact of IQ and task complexity on the processing of targets and lures. Data were excluded if the response to a target or lure was incorrect and were from occasional chunks with poor overall performance (see Materials and Methods).

**Figure 3.**
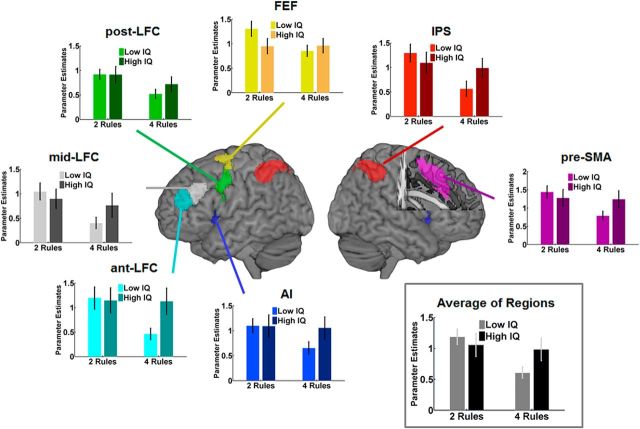
fMRI results from multiple-demand network ROIs. Graphs plot the mean parameter estimates for targets and lures, separately for low IQ and high IQ groups during two-rule and four-rule conditions, averaged across hemispheres and stimulus types. The gray box presents the average of activation across all presented ROIs. Error bars indicate SEM.

**Figure 4.**
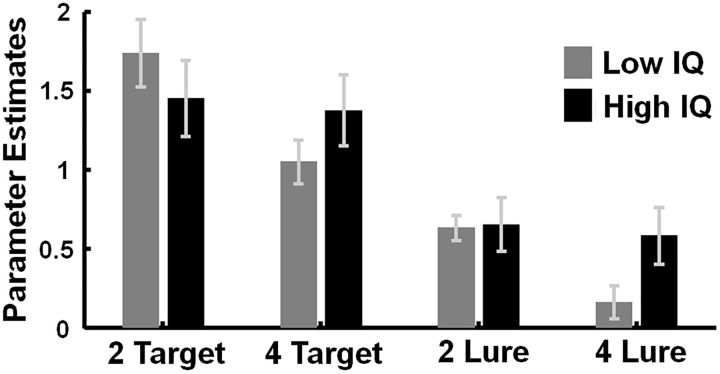
fMRI results from multiple-demand network ROIs. Mean parameter estimates across all ROIs for low-IQ and high-IQ groups during two-rule and four-rule conditions, plotted for targets and lures separately. Error bars indicate SEM.

An ANOVA was applied to parameter estimates of targets and lures. This included the factors complexity (two-rule vs four-rule runs), IQ group (low-IQ vs high-IQ), “stimulus” (targets vs lure), “hemisphere” (left vs right), and ROI (ant-LFC, mid-LFC, post-LFC, FEF, IPS, AI, and pre-SMA). Importantly, a main effect of complexity (*F*_(1,34)_ = 12.75, *p* = 0.001) was observed, as well as a significant complexity × IQ group interaction (*F*_(1,34)_ = 7.16, *p* = 0.011). These effects did not interact with any other factor. Figure 3 shows mean parameter estimates averaged across stimuli (targets, lures) and hemispheres, separately for each ROI and the average across ROIs. The results show that the main effect of complexity was driven by smaller parameter estimates for the more challenging four-rule runs, compared with the two-rule runs. The complexity × IQ group interaction was due to particularly low parameter estimates in the low-IQ group on four-rule runs. No significant stimulus × complexity × IQ group interaction was observed, suggesting a similar response pattern for targets and lures (Fig. 4).

**Figure 5.**
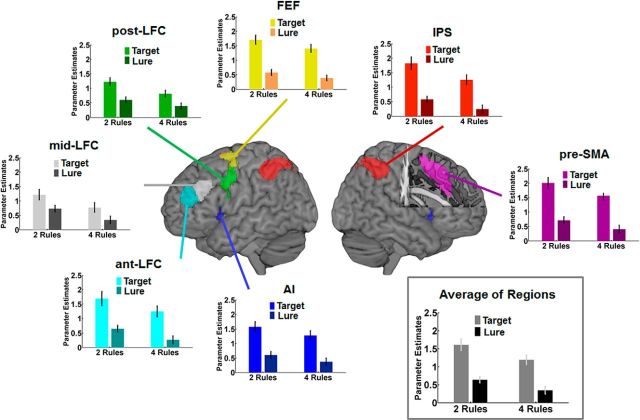
fMRI results from multiple-demand network ROIs. Graphs plot the mean parameter estimates of all participants for targets and lures during two-rule and four-rule conditions, averaged across hemispheres. The gray box presents the average of activation across all presented ROIs. Error bars indicate SEM.

A strong main effect of stimulus was also observed (*F*_(1,34)_ = 68.39, *p* < 0.001), reflecting an overall difference between targets and lures (Fig. 5). The size of this difference varied somewhat across ROIs, revealed in significant interactions of stimulus × ROI (*F*_(1,29)_ = 30.62, *p* < 0.001), stimulus × hemisphere × ROI (*F*_(1,29)_ = 7.38, *p* < 0.001), and stimulus × hemisphere × IQ group (*F*_(1,34)_ = 4.41, *p* = 0.043). Overall, however, the strong trend was simply for greater response to targets than lures.

Effects of IQ group and complexity were also explored for cues and filler items. ANOVAs were applied to parameter estimates from multiple-demand regions for each of these stimulus types. However, no significant main effect of complexity and no significant complexity × IQ group interaction were observed for either cues (all *p* values >0.481) or fillers (all *p* values >0.363). Thus, the main impact of complexity and IQ group concerned only responses to the two specific, task-critical events, the current target (currently cued) and lure (cued in other task periods).

**Figure 6.**
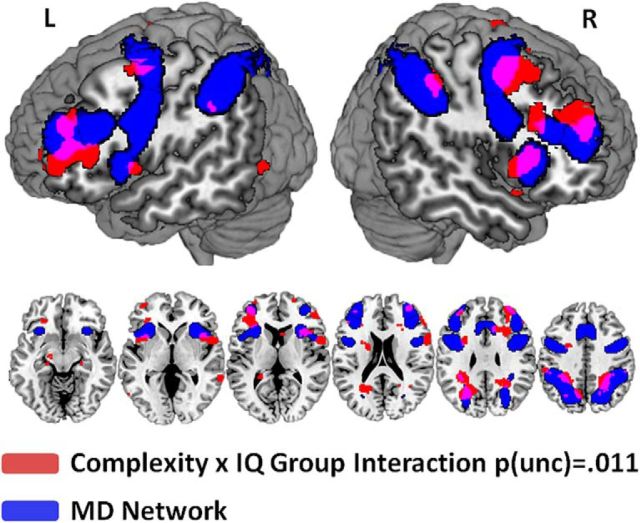
Overlap of multiple-demand network region masks (blue) with complexity × IQ group interaction from a whole-brain analysis on targets and lures.

Finally, to check for activity outside our a priori ROIs, we ran a second-level whole-brain ANOVA on parameter estimates from targets and lures. This analysis included the factors complexity (two-rule vs four-rule runs), IQ group (low-IQ vs high-IQ), and stimulus (targets vs lures). No false discovery rate-corrected significant complexity or IQ group effects were observed at the whole-brain level. At a lenient threshold of *p*(uncorrected) = 0.011, which was the level of significance of the complexity × IQ group interaction in ROI analyses, this interaction only revealed effects within or adjacent to regions of the multiple-demand network (Fig. 6). The results confirm that it was multiple-demand network regions that were most affected by our experimental manipulations.

## Discussion

This study addressed the neural basis of human fluid intelligence and its link to the demands of rule use. Effects of fluid intelligence under varying task demands were observed in both behavioral performance as well as in neural measures extracted from a specific set of frontal and parietal regions, previously defined as the multiple-demand network (Duncan, 2010; Fedorenko et al., 2013). Task demands were manipulated by introducing smaller and larger sets of novel rules across experimental runs (two vs four rules). Within each run, only one rule was cued at a time in a continuous sequence of stimuli, keeping the complexity of each single task period constant across runs. Participants from low-IQ and high-IQ groups were instructed to respond to the cued target and to ignore the images associated with a different cue (lure) as well as those never associated with any cue (fillers). Accordingly, complexity differences between two-rule and four-rule runs reflected the difficulty of foregrounding and implementing the specific rule of the current task period, when this was to be drawn from a smaller or larger overall set.

We observed a main effect of complexity (two-rule vs four-rule runs) in behavioral performance accuracies and neural signal from multiple-demand network regions. Participants showed weaker neural signal in four-rule runs in contrast to two-rule runs for task-critical events (targets and lures), accompanied by lower performance accuracies (more missed targets) on four-rule runs. Neural responses to fillers did not show these effects, thus providing evidence that those effects are not due to low-level fluctuations in neural signal across experimental runs. Together, this indicates a less focused use of task rules in the case of a larger task set. Importantly, complexity interacted with fluid intelligence on both the behavioral and neural level for critical task events (targets and lures). Low-IQ participants showed particularly high error rates on four-rule runs and weaker neural signal across all multiple-demand network regions, which speaks for a closely integrated network. Whole-brain analyses confirmed the localization of these effects within, or adjacent to, regions of the multiple-demand network. Follow-up research with increased subject samples and thus increased statistical power may reveal fluid intelligence-dependent rule representations within additional frontal structures (compare with Sakai and Passingham, 2006; Reverberi et al., 2012).

In a related prior study, Gray et al. (2003) linked frontoparietal activity to fluid intelligence in a three-back working memory task. In their data, as in ours, high fluid intelligence was associated with stronger frontoparietal responses to challenging task events (lure stimuli that had appeared not three places back in the sequence, but nearby). Our results extend this finding to a new task domain, address the role of task complexity, and show similar activity across the whole distributed multiple-demand network. More broadly, a complex literature relates individual differences in cognitive ability to activation of frontoparietal cortex, with some findings showing greater activity for high-ability participants and others showing the reverse (Reuter-Lorenz et al., 2000; Rypma and D'Esposito, 2000; Cabeza, 2002; Szameitat et al., 2016). In aging, for example, stronger recruitment for better-performing individuals has been linked to compensation for cognitive decline (Reuter-Lorenz et al., 2000; Cabeza, 2002), while in other cases the reverse result may reflect increased cognitive demand for low-performing individuals. In our case, as in the study of Gray et al. (2003), the critical data concern the response to specific, task-critical events. In this case, we suggest that weak recruitment in low-ability individuals reflects poor attentional focus or poor discrimination of critical events from the ongoing task background. A similar argument may explain why, in our study, it was the harder four-rule task that was associated with reduced multiple-demand activity, in contrast to the common finding of increased activity with increasing task difficulty (Duncan and Owen, 2000; Fedorenko et al., 2013).

Our results are consistent with previous research reporting a phenomenon of goal neglect in individuals with lower fluid intelligence (Duncan et al., 2008; Bhandari and Duncan, 2014). In goal neglect, task requirements are repeatedly ignored during performance, although subjects accurately describe them before and after testing. Although fluid intelligence correlates with a range of traditional working memory measures that capture task execution demands such as digit span, spatial span, and visual short-term memory (Engle et al., 1999; Kane and Engle, 2002; Fukuda et al., 2010), particularly high correlations have been observed with the neglect of novel task rules (Duncan et al., 2012). Goal neglect increases with the number of rules in a task (Duncan et al., 2008) and has been linked to difficulty in focusing on the specific rules required in a current task step (Bhandari and Duncan, 2014). Our data suggest similar processing limits even in the absence of major performance failures and link them to a reduced response to critical task events across the multiple-demand network, especially in individuals with low fluid intelligence.

To conclude, this study demonstrated a close link between multiple-demand network functions and a core feature of fluid intelligence: the process of dividing complex tasks in a sequence of attentional episodes. Thus, the multiple-demand network may orchestrate the allocation of attentional resources to individual parts of a complex task. In high fluid intelligence, more attentional resources are allocated to those parts of a task that are most critical for behavior.
